# Ten myths of back pain in older adults that can lead to ineffective and harmful care

**DOI:** 10.1186/s12998-025-00609-9

**Published:** 2025-10-15

**Authors:** Carlo Ammendolia

**Affiliations:** 1 Department of Surgery, University of Toronto, Toronto, Canada; 2 Department of Anesthesiology and Pain Management, Mount Sinai Hospital, Toronto, Canada

**Keywords:** Back pain, Older adults, Myths, Ineffective care, Harms

## Abstract

Low back pain (LBP) is one of the most disabling conditions in older adults and among the costliest in terms of healthcare expenditures. Many factors contribute to the disability and high costs of LBP in older adults, but one of the most preventable is the spread of misinformation and unhelpful attitudes, beliefs, and behaviors. These are often perpetuated by family, friends, social media, pharmaceutical companies, other industries, and healthcare providers. Myths about back pain foster false attitudes, beliefs, and behaviors that lead to inappropriate, costly, and sometimes harmful treatments. Such myths can result in psychological consequences, including fear of movement, poor self-efficacy, low motivation, anxiety, stress, and depression- all of which further perpetuate disability. Injections, surgeries, and medications for non-specific LBP are usually ineffective and are associated with significant side effects in older adults. The purpose of this paper is to dispel ten common myths of LBP in older adults, with the goals of changing attitudes, beliefs, and behaviors to reflect a more positive and evidence-based approach among practitioners and public. The aim is also to motivate practitioners to educate their older patients based on the best available evidence. This can improve outcomes, reduce costs, reduce disability, and improve quality of life among older adults with back pain.

## Introduction

Low back pain (LBP) is one of the most disabling health conditions in older adults and one of the costliest in terms of healthcare expenditures [1, 2]. Many factors contribute to the disability and high costs of LBP in older adults, but one of the most preventable is misinformation and unhelpful attitudes, beliefs, and behaviors perpetuated by family, friends, social and traditional media, pharmaceutical companies, other industries, and healthcare providers [3–7]. Several papers and editorials have been written on the myths of back pain in the general population [6–8] but none focus specifically on older adults, usually defined as age 65 years or older [9]. Back pain in older adults is more complex and may therefore be more prone to misconception and misinformation, which can lead to inappropriate care [10]. The purpose of this paper is to dispel ten current common myths of LBP in older adults, with the goals of changing attitudes, beliefs, and behaviors of healthcare practitioners to align with best- evidence care for this population. This can lead to reduced costs and improved patient outcomes [11].

## Main text

### Methods

This paper presents a clinical expert commentary grounded in the author's extensive experience in clinical practice and research related to low back pain in older adults. A targeted literature search was conducted to identify evidence supporting the author’s statements and recommendations. The search included peer-reviewed sources such as PubMed, Google Scholar, and the Cochrane Library covering publications to April 15th, 2025. Key search terms included "low back pain," "older adults," "myths," "misinformation," and "ineffective treatments".

## Results

### Myths of back pain in older adults. (See Table 1 for key messages).

**Table 1:** Take Home messages for clinical practice

Myth	Fact
1. Back pain is inevitable with aging	Common but not inevitable; Prevalence levels off after age 60 [12–14]
2. Back pain usually indicates serious disease in older adults	Serious conditions < 5%; most cases are non-specific [14–17]
3. Imaging is necessary in adults > 50 with LBP	Imaging without red flags can cause more harm than good [15, 18–22]
4. Let the pain guide you. Avoid lifting, twisting and bending with LBP	Activity promotes recovery; inactivity worsens outcomes. Pain with activity does not usually cause harm [23–28]
5. Bed rest is recommended for back pain in older adults	Bed rest can cause more harm than good for back pain [23, 29]
6. Medication should be first-line treatment	Non-pharmacological treatments recommended as first line treatment [25, 30–35]
7. Surgery is effective for back dominant primary back pain	Surgery not recommended for primary back-dominant pain and can cause more harm than good [29, 36–42]
8. Chronic LBP in older adults is always due to structural damage	Structural changes poorly corelates with presence of back pain. Psychosocial factors play major role [12, 14, 15, 26, 28, 44–47]
9. Injections, ablation and nerve blocks are highly effective	Benefits no better than sham treatment with increased adverse events in older adults [22, 48–51]
10. Disc herniations commonly cause leg pain in older adults	Less common; clinical signs more reliable than imaging [19, 52, 53]

1. Myth: Back pain is inevitable as we age and is part of the normal aging process.

*Fact* Back pain is common as we age, but it is not inevitable. Once serious diseases such as cancer, infection, or fracture are ruled out, the prevalence of non-specific (also known as primary) back pain increases until about age 60, then levels off and can slightly decrease [12, 13]. Research suggests that working-age adults are most at risk for low back pain, which lessens in older post-retirement age groups [14]. However, the severity of back pain and risk of chronic LBP and disability increases with age [12]. This may be due to the increased prevalence of specific causes of back pain in older adults, such as osteoarthritis, spinal stenosis, and osteoporosis, which are associated with higher levels of chronic pain and disability [12]. Resources may be better spent on early identification and treatment of specific causes of LBP in older adults.

2. Myth: Back pain in older adults is usually due to a serious medical problem.

*Fact* Although serious diseases such as cancer, fracture, and infection are more common causes of LBP in older adults, they represent less than 5% of all causes of LBP in this age group [14]. Primary (or non-specific) LBP is the most common type of LBP across all age groups, including older adults, accounting for approximately 90% of all cases [14, 15]. Other serious secondary causes of back pain include cauda equina syndrome (CES) and inflammatory back pain, neither of which are more common in older adults. The peak age for CES is between 30 and 40 years [16], and for inflammatory back pain, such as ankylosing spondylitis, onset usually occurs in late adolescence and early adulthood, with the most common onset before age 40 [17].

3. Myth: People over the age of 50 who present with LBP should undergo imaging to rule out serious disease and identify the cause of their pain.

*Fact* In the absences of red flags suggestive of serious disease, imaging is not recommended [15]. While age over 50 has been listed as a red flag in some clinical practice guidelines for LBP, it is not intended to be used in isolation. Instead, it should be considered alongside other red flags, such as a previous history of cancer [18]. Routine imaging for LBP, including in older adults, is not advised. In most cases, LBP in older adults is classified as primary or non-specific, and imaging rarely identifies the source of pain [15]. Abnormal imaging findings are common in asymptomatic older adults, such as degenerative facet joints, thinning and bulging discs, narrowed central and lateral spinal canals, thickening of the ligamentum flavum, and neural compression [19]. Even among symptomatic individuals, the degree of degenerative changes typically does not correlate with the severity of symptoms. Imaging findings in older adults are rarely useful in predicting outcomes in primary back pain [20, 21]. Incidental findings on unnecessary imaging can lead to further unwarranted testing and specialist referrals, which may cause psychological stress and result in poorer outcomes [15, 22].

4. Myth: Older adults with back pain and should avoid lifting heavy objects, twisting or bending activities. Let the pain guide them- if it hurts, don't do it.

*Fact* Older adults who present without red flags and have back-dominant primary LBP should be advised to continue with their normal activities despite the pain. There is no evidence that pain experienced during activity in cases of non-specific LBP causes damage to the lower back [23]. In fact, pain avoidance behaviors, including avoiding exercise, delays recovery [24]. This applies to degenerative or osteoarthritic related back pain where "motion is lotion" - activity can reduce back pain with no evidence that activity worsens the osteoarthritis [25]. Activities requiring lifting, twisting and bending are beneficial for maintaining a healthy back. Avoiding such movements can lead to increased pain, stiffness and physical deconditioning [26]. An older spine is not a contraindication to these activities and is not at greater injury risk compared to a younger spine [27]. Most back pain in older adults is not caused by injury [28]. Graduated lifting and progressive physical demands to the spine help build structural resilience and overall fitness [26]. Conversely, fear associated with such activities can lead to avoidance behaviours and reduced self-efficacy, increasing the risk for fear-related back disability [28].

5. Myth: Bed rest is recommended for back pain among older adults.

*Fact* Bed rest is particularly discouraged for older adults with back pain. In addition to evidence showing that bed rest prolongs recovery [23], older adults are at higher risk of negative consequences of bed rest including increase joint stiffness, muscle wasting, loss of bone mineral density, pressure ulcers and venous thromboembolism [29]. Maintaining active lifestyle despite back pain will reduce risk of *homeostenosis,* which is defined as the progressive restriction of the ability to respond to stress with aging [29]. Staying physically active and incorporating regular aerobic and strength training exercises helps preserve muscle reserves needed to maintain function as we age [29].

6. Myth: Medication should be the first-line treatment for older adults with LBP.

*Fact* First-line treatment for primary LBP should be non-pharmacological. Acetaminophen, a commonly prescribed drug, has been shown to be no better than placebo in high quality adult trials [25]. NSAIDS, also are commonly prescribed, offer only small benefits and should be avoided in older adults who are on blood thinners, have high blood pressure or at risk for gastric bleeding [25]. Muscle relaxants can cause light-headedness and should be avoided in older adults with balance issues [25]. Antiseizure drugs like pregabalin and gabapentin are often prescribed for neuropathic- related back pain but have been shown to be no better than placebo and associated with dizziness and cognitive impairment [31–33], which can negatively impact older adults with risk of falls. While certain antidepressant may offer minimal pain relief for back pain in older adults, the benefits are generally modest and may not outweigh the risks, which include, cardiotoxicity, dizziness, weight gain, decrease bone density and increase risk of falls [34, 35]. There is also the risk of negative drug interaction among older adults taking multiple medications [35].

7. Myth: Surgery is effective for back-dominant pain in older adults.

*Fact* Surgery for primary back-dominant pain (without lower extremity radicular symptoms) is not supportive by current evidence and potentially can cause more harm than good in older adults [36–38]. Older adults often present with multiple comorbidities making them more susceptible to surgical complications [38]. Complication rates for spinal surgeries in older adults range from 3% to 29%, with higher risks associated with advanced age [39].

Despite the lack of evidence and increased risks, there has been a substantial rise in spine surgery for LBP in older adults [38, 40, 41]. A key factor driving this increase is the growing number of individuals over age 65 presenting with chronic LBP and degenerative findings on advanced imaging, who are subsequently referred for surgical consultations [29, 38]. However, degenerative imaging findings become more common with age and correlates poorly with presence of LBP [42].

There is evidence supporting back surgery for leg- dominant radicular symptoms, such as from a herniated disc or lumbar spinal stenosis [43]. Lumbar spinal stenosis is the most common reason for spine surgery in individuals over age 65 [43]. However, even among patients who undergo surgery for radicular symptoms, including lumbar spinal stenosis, their LBP often does not improve [37].

8. Myth: Chronic LBP in older adults is always due to structural damage.

*Fact* Almost all older adults have degenerative changes of the lumbar spine, yet only a small fraction experience chronic LBP, and even fewer are disabled by it [12, 14]. Searching for a physical cause of non-specific chronic LBP is rarely productive and often leads to expensive and unnecessary imaging, other tests and referrals, which can worsen pain and disability [15]. Primary, or non-specific, chronic LBP is a biopsychosocial condition. It may begin with a physical issue, but the pain persists beyond the usual healing time and in the absence of significant physical findings [44, 45]. The strongest predictors of chronic LBP in older adults are psychosocial factors, including fear avoidance behaviour, poor self-efficacy, low motivation and low mood [28, 46, 47]. There may be physical consequences in older adults with chronic LBP that are secondary to fear of movement, such as deconditioning, weight gain, bone loss, and sarcopenia. However, successful care of chronic LBP in older adults requires a multimodal approach focused on addressing psychosocial factors and maladaptive behaviours and beliefs [26, 45]

9. Myth: Interventional procedures such as epidural injections, joint injections, nerve blocks or radiofrequency ablations are effective in older adults with chronic back pain.

*Fact* Recent evidence suggests that these interventional procedures are no better than sham procedures for back or radicular pain of more than three months [48, 49]. These procedures are commonly prescribed when conservative treatments have failed, and back pain persists. However, despite the lack of evidence of effectiveness supporting their effectiveness beyond placebo, the use of these procedures continues to grow and is associated with high costs [22, 49]. There is an increased risk of adverse events in older adults receiving these procedures, including hyperglycemia and bone mineral loss with steroid injections [50], and temporary motor weakness with nerve blocks [51]. These side effects can increase the risk of falls and fractures in this population [50].

10. Myth: Disc herniations causing back- related leg pain and disability are very common in older adults

*Fact* The peak age for symptomatic disc herniations is between 30-50 years, and they become less likely to be a direct source of nerve compression causing leg pain with advancing age [52]. This is due to gradual disc desiccation, which leads to fluid loss and eventual stiffening or calcification of the disc [53]. Although, imaging in older adults can demonstrate herniated or bulging discs, these typically represent old and asymptomatic herniations or bulges that have calcified and become rigid and static. They typically do not displace with activities such as bending forward or sitting [19]. It is important to interpret imaging findings in older adults with caution and to rely more on clinical signs of nerve tension and neurological deficits as reliable indicators of radiculopathy across all age groups.

### Clinical implications

The myths surrounding LBP in older adults contribute significantly to the perpetuation of ineffective and potentially harmful treatments. These misconceptions can lead to inappropriate clinical practices and exacerbate both the psychological and physical burden on older adults [10]. The consequences of these myths extend beyond clinical mismanagement. They can influence contemporary health policy, often leading to over-reliance on imaging, interventional procedures, surgical interventions, and pharmacologic treatments that lack evidence for efficacy in this population [11]. In care delivery, myths contribute to ageist assumptions, underutilization of conservative therapies, and fragmented interdisciplinary approaches [54]. Media and social media further amplify these myths, frequently portraying aging spines as inherently pathological and promoting fear-based narratives that reinforce avoidance behaviors and passive coping strategies [55].

To address these myths, a multi-pronged strategy is needed. First, educational campaigns and credible online resources should target both clinicians and the public, emphasizing evidence-based messages about aging and spinal health [56]. Institutions training health care practitioners who treat older adults with back pain should critically assess their curricula to ensure instruction is grounded in the best available evidence and free from myths and misinformation.

Second, clinical guidelines must be updated to reflect the nuanced presentation of LBP in older adults, discouraging interventions that are not supported by sound evidence. Guidelines should also provide clearer diagnostic frameworks that distinguish between age-related physiological changes and pathological conditions, reducing the risk of overmedicalization.

Finally, policy makers should be encouraged to fund and promote conservative models of care in older adults that prioritize function, patient engagement, and biopsychosocial approaches.

Future research should examine the impact of myth-driven care on functional outcomes and quality of life in older populations with LBP. Researchers should also investigate the role of digital media in shaping beliefs about spine health in older adults and develop tools to counter misinformation. Addressing these gaps will be essential for improving care and outcomes for this growing demographic.

A key limitation of this paper is its reliance on a single expert’s perspective and the selection of supporting evidence that may reflect the author’s bias. Despite this, the findings and recommendations in this paper align well with recent World Health Organization guidelines for LBP [15]. Another limitation is the lack of standardized definitions for "older adults" in the literature which may complicate the generalizability of findings.

## Conclusions

The persistence of myths about LBP in older adults represents a critical barrier to effective, evidence-based care. These misconceptions distort clinical decision-making, public perception, policy development, and media narratives in ways that promote unnecessary and sometimes harmful interventions. There is a dire need to dispel these myths and to do so we need unified, coordinated and multipronged approaches across education, clinical practice, policy and research.

## Data Availability

No datasets were generated or analysed during the current study.
