# Correction: Ten myths of back pain in older adults that can lead to ineffective and harmful care

**DOI:** 10.1186/s12998-026-00638-y

**Published:** 2026-03-30

**Authors:** Carlo Ammendolia

**Affiliations:** 1 Department of Surgery, University of Toronto, Toronto, Canada; 2 Department of Anesthesiology and Pain Management, Mount Sinai Hospital, Toronto, Canada

**Correction: Chiropractic & Manual Therapies (2025) 33:45** 10.1186/s12998-025-00609-9

The author apologizes to the readers for errors in the reference list of the original publication. ChatGPT was used to assist with formatting references in Vancouver style, which led to the inclusion of fictitious references that the author failed to verify prior to submission. The article content & conclusions remain unchanged.

Furthermore, there was 1 citation error:

- Under myth #6 citation [20] should have been citation [25], this reference was present in the original publication.

The incorrect and correct references are shown in this correction article, the original article has been updated.

**Table:** 

**Correct references**	**Incorrect references**
1. Vos T, Abajobir AA, Abate KH, Abbafati C, Abbas KM, Abd-Allah F, Abdulkader RS, Abdulle AM, Abebo TA, Abera SF, Aboyans V. Global, regional, and national incidence, prevalence, and years lived with disability for 328 diseases and injuries for 195 countries, 1990–2016: a systematic analysis for the Global Burden of Disease Study 2016. The Lancet. 2017 Sep 16;390(10100):1211-59.	1. Vos T, Abajobir AA, Abate KH, Abbafati C, Abbas KM, Abd-Allah F, et al. Global, regional, and national incidence, prevalence, and years lived with disability for 310 diseases and injuries, 1990–2016: a systematic analysis for the global burden of disease study 2016. Lancet. 2017;390:1211–59.
2. Dieleman JL, Cao J, Chapin A, Chen C, Li Z, Liu A, Horst C, Kaldjian A, Matyasz T, Scott KW, Bui AL. US health care spending by payer and health condition, 1996-2016. Jama. 2020 Mar 3;323(9):863-84	2. Dieleman JL, Cao J, Chapin A, Chen C, Li Z, Liu A, et al. US health care spending by payer and health condition, 1996–2016. JAMA. 2020;323(9):863–84.
3. Buchbinder R, van Tulder M, Öberg B, Costa LM, Woolf A, Schoene M, Croft P, Hartvigsen J, Cherkin D, Foster NE, Maher CG. Low back pain: a call for action. The Lancet. 2018 Jun 9;391(10137):2384-8.	3. Buchbinder R, van Tulder M, Öberg B, Costa LM, Woolf A, Schoene M, et al. Low back pain: a call for action. Lancet. 2018;391(10137):2384–8.
4. Hartvigsen J, Hancock MJ, Kongsted A, Louw Q, Ferreira ML, Genevay S, Hoy D, Karppinen J, Pransky G, Sieper J, Smeets RJ. What low back pain is and why we need to pay attention. The Lancet. 2018 Jun 9;391(10137):2356-67.	4. Hartvigsen J, Hancock MJ, Kongsted A, Louw QA, Ferreira ML, Genevay S, et al. What low back pain is and why we need to pay attention. Lancet. 2018;391(10137):2356–67.
5. Foster NE, Anema JR, Cherkin D, Chou R, Cohen SP, Gross DP, Ferreira PH, Fritz JM, Koes BW, Peul W, Turner JA. Prevention and treatment of low back pain: evidence, challenges, and promising directions. The Lancet. 2018 Jun 9;391(10137):2368-83	5. Foster NE, Anema JR, Cherkin D, Chou R, Cohen SP, Gross DP, et al. Prevention and treatment of low back pain: evidence, challenges, and promising directions. Lancet. 2018;391(10137):2368–83.
6. Deyo RA. Low-back pain. Sci Am. 1998 Aug;279(2):48-53.	6. Deyo RA. Back pain revisited: a 21st century perspective. Sci Am. 1998;279(2):48–53.
7. O'Sullivan PB, Caneiro JP, O'Sullivan K, Lin I, Bunzli S, Wernli K, O'Keeffe M. Back to basics: 10 facts every person should know about back pain. British journal of sports medicine. 2020 Jun 1;54(12):698-9.	7. O’Sullivan PB, Caneiro JP, O’Keeffe M, O’Sullivan K. Back to basics: 10 facts every person should know about back pain. Br J Sports Med. 2020;54(12):698–702.
8. Kripa S, Kadiresan M. Low back pain myths: a narrative review. Bulletin of Faculty of Physical Therapy. 2024 Oct 31;29(1):62.	8. Honda R, Matsudaira K, Sawada T, Isomura T, Ishida T. Ten commonly held beliefs about low back pain. J Orthop Sports Phys Ther. 2019;49(11):842–7.
9. National Institutes of Health (NIH). Age [Internet]. Bethesda (MD): NIH; [cited 2025 Aug 26]. Available from: https://www.nih.gov/nih-style-guide/age	9. National Institutes of Health (NIH). Age [Internet]. Bethesda (MD): NIH; [cited 2025 Aug 26]. Available from: https://www.nih.gov/nih-style-guide/ageNIH
10. Weiner DK, Marcum Z, Rodriguez E. Deconstructing chronic low back pain in older adults: Summary recommendations. Pain Medicine. 2016 Dec 1;17(12):2238-46.	10. Weiner DK, Marcum Z, Rodriguez E. Deconstructing chronic low back pain in older adults: summary recommendations. Pain Med. 2016;17(12):2238–46. https://doi.org/10.1093/pm/pnw267.
11. Docking S, Sridhar S, Haas R, Mao K, Ramsay H, Buchbinder R, O'Connor D. Models of care for managing non‐specific low back pain. Cochrane Database of Systematic Reviews. 2025(3).	11. Docking S, Sridhar S, Haas R, Mao K, Ramsay H, Buchbinder R, et al. Models of care for managing non-specific low back pain. Cochrane Database Syst Rev. 2025. https://doi.org/10.1002/14651858.CD015083.pub2.
12. Dionne CE, Dunn KM, Croft PR. Does back pain prevalence really decrease with increasing age? A systematic review. Age and ageing. 2006 May 1;35(3):229-34	12. Dionne CE. Predictors of “frequent” and “chronic” low back pain in the general population. Spine. 2006;31(22):2479–87.
13. De Souza IM, Sakaguchi TF, Yuan SL, Matsutani LA, do Espírito-Santo AD, de Braganca Pereira CA, Marques AP. Prevalence of low back pain in the elderly population: a systematic review. Clinics. 2019 Jan 1;74:e789.	13. De Souza IMB, Sakaguchi TF, Yuan SLK, Matsutani LA, do Espírito-Santo AS, Pereira CAB, et al. Prevalence of low back pain in older adults: a systematic review. Eur Spine J. 2019;28(4):716–29.
14. Docking R, Fleming J, Brayne C, Zhao J, Macfarlane J, Jones T. Epidemiology of the back pain in older adults. prevalence and risk factors for back pain onset. Rheumatology (Oxford). 2011;50(9):1645-53.	14. Docking RE, Fleming J, Brayne C, Zhao J, Macfarlane GJ, Jones GT. Epidemiology of back pain in older adults. Age Ageing. 2011;40(6):735–41.
15. World Health Organization. WHO guideline for non-surgical management of chronic primary low back pain in adults in primary and community care settings. World Health Organization; 2023 Dec 7.	15. World Health Organization. Low back pain guidelines. Geneva: WHO; 2023.
16. Woodfield J, Lammy S, Jamjoom AA, Fadelalla MA, Copley PC, Arora M, Glasmacher SA, Abdelsadg M, Scicluna G, Poon MT, Pronin S. Demographics of cauda equina syndrome: a population-based incidence study. Neuroepidemiology. 2022 Dec 28;56(6):460-468.	16. Woodfield J, Adams S, Siddiqui A, Morcos C. Cauda equina syndrome in older adults. Spine J. 2022;22(4):789–97.
17. Lassiter W, Bhutta BS, Allam AE. Inflammatory back pain. InStatPearls [Internet] 2024 Feb 26. Statpearls Publishing.	17. Lassiter W, Edwards M, Nash P, Maddison P. Age-related onset of inflammatory back pain. Curr Rheumatol Rep. 2024;26(1):12–9.
18. Koes BW, Van Tulder MW, Ostelo R, Burton AK, Waddell G. Clinical guidelines for the management of low back pain in primary care: an international comparison. Spine. 2001 Nov 15;26(22):2504-13.	18. Koes B, van Tulder M, Lin CW, Macedo LG, McAuley J, Maher C. Red flags for back pain: are they useful? Spine. 2001;26(9):990–5.
19. Boden SD, Davis DO, Dina TS, Patronas NJ, Wiesel SW. Abnormal magnetic-resonance scans of the lumbar spine in asymptomatic subjects. A prospective investigation. J Bone Joint Surg Am. 1990 Mar;72(3):403-8.	19. Boden SD, Davis DO, Dina TS, Patronas NJ, Wiesel SW. Abnormal magnetic-resonance scans of the lumbar spine in asymptomatic subjects. J Bone Joint Surg Am. 1990;72(3):403–8.
20. Steffens D, Hancock MJ, Maher CG, Williams C, Jensen TS, Latimer J. Does magnetic resonance imaging predict future low back pain? A systematic review. European journal of pain. 2014 Jul;18(6):755-65.	20. Steffens D, Hancock MJ, Maher CG, Williams CM, Jensen TS, Latimer J. Imaging findings and their relationship with clinical outcomes in low back pain. Eur J Pain. 2014;18(3):383–93.
21. Maiers MJ, Sundin AR, Oster RJ, Kreul S, Malone Q, Passmore SR. Contextual factors related to aging determine force-based manipulation dosage: a prospective cross-sectional study. Chiropractic & Manual Therapies. 2025 May 21;33(1):20.	21. Maiers MJ, Sundin AR, Kreul S. Contextual factors related to aging determine force-based manipulation dosage: a prospective cross-sectional study. Chiropr Man Therap. 2025;33(1):18. https://doi.org/10.1186/s12998-025-00584-1.
22. Powell AC, Rogstad TL, Elliott SW, Price SE, Long JW, Deshmukh UU, Murad MH, Steffen MW. Health care utilization and pain outcomes following early imaging for low back pain in older adults. The Journal of the American Board of Family Medicine. 2019 Nov 1;32(6):773-80.	22. Powell AC, Rogstad TL, Elliott SW, Price SE, Long JW, Deshmukh UU, et al. Health care utilization and pain outcomes following early imaging for low back pain in older adults. J Am Board Fam Med. 2019;32(6):773–80. https://doi.org/10.3122/jabfm.2019.06.190103.
23. Vadalà G, Russo F, De Salvatore S, Cortina G, Albo E, Papalia R, Denaro V. Physical activity for the treatment of chronic low back pain in elderly patients: a systematic review. Journal of clinical medicine. 2020 Apr 5;9(4):1023.	23. Vadala G, Russo F, De Salvatore S, Corona F, Bonifacino E, Muratore M, et al. Motion is lotion: keeping older backs healthy. Aging Res Rev. 2020;61:101112.
24. Hill JC, Dunn KM, Lewis M, Mullis R, Main CJ, Foster NE, Hay EM. A primary care back pain screening tool: identifying patient subgroups for initial treatment. Arthritis Care & Research: Official Journal of the American College of Rheumatology. 2008 May 15;59(5):632-41.	24. Hill JC, Dunn KM, Lewis M, Mullis R, Main CJ, Foster NE. A primary care back pain screening tool: identifying patient subgroups for initial treatment. Arthritis Rheum. 2008;59(5):632–41. https://doi.org/10.1002/art.23563.
25. Cashin AG, Wand BM, O'Connell NE, Lee H, Rizzo RR, Bagg MK, O'Hagan E, Maher CG, Furlan AD, van Tulder MW, McAuley JH. Pharmacological treatments for low back pain in adults: an overview of Cochrane Reviews. Cochrane Database of Systematic Reviews. 2023(4).	25. Cashin AG, Folly T, Oliveira CB, Courtney C, Poitras S, Lin CC, et al. Acetaminophen for low back pain: a systematic review and meta-analysis. BMJ. 2023;372:m4825.
26. O’Sullivan PB, Caneiro JP, O’Keeffe M, Smith A, Dankaerts W, Fersum K, O’Sullivan K. Cognitive functional therapy: an integrated behavioral approach for the targeted management of disabling low back pain. Physical therapy. 2018 May;98(5):408-23.	26. O’Sullivan PB, Caneiro JP, O’Keeffe M, Smith A, Dankaerts W, Fersum K, et al. Cognitive functional therapy: an integrated behavioral approach for the targeted management of disabling low back pain. Phys Ther. 2018;98(5):408–23.
27. Mauri Kallinen AM. Aging, physical activity and sports injuries. Sports Medicine. 1995;20(1):41-52.	27. Kallinen M, Markku A. Aging, physical activity and sports injuries. Scand J Med Sci Sports. 1995;5(1):20–4.
28. Wong AY, Karppinen J, Samartzis D. Low back pain in older adults: risk factors, management options and future directions. Scoliosis and spinal disorders. 2017 Apr 18;12(1):14.	28. Wong AYL, Karppinen J, Samartzis D. Low back pain in older adults: risk factors, management options and future directions. Scoliosis Spinal Disord. 2017;12:14.
29. Weiner DK. Deconstructing chronic low back pain in the older adult: Shifting the paradigm from the spine to the person. Pain Medicine. 2015 May 1;16(5):881-5.	29. Weiner DK, Rudy TE, Morrow L, Slaboda J, Lieber S. Pain management in the elderly: bridging the gap. Pain Med. 2015.
30. McCarberg BH. NSAIDs in the older patient: balancing benefits and harms. Pain Medicine. 2013 Dec 1;14(suppl_1):S43-4.	30. McCarberg BH. Nsaids in the management of low back pain. J Pain Res. 2013;6:251–67.
31. Enke O, New HA, New CH, Mathieson S, McLachlan AJ, Latimer J, Maher CG, Lin CW. Anticonvulsants in the treatment of low back pain and lumbar radicular pain: a systematic review and meta-analysis. Cmaj. 2018 Jul 3;190(26):E786-93.	31. Enke O, New HA, Gilbert EA, Whittaker S, Huang Z, Siersma VD, et al. Antiseizure medications for chronic non-cancer pain: a Cochrane review. Cochrane Database Syst Rev. 2018;8:CD01567.
32. Shanthanna H, Gilron I, Rajarathinam M, AlAmri R, Kamath S, Thabane L, Devereaux PJ, Bhandari M. Benefits and safety of gabapentinoids in chronic low back pain: a systematic review and meta-analysis of randomized controlled trials. PLoS medicine. 2017 Aug 15;14(8):e1002369.	32. Shanthanna H, Busse JW, Ebrahim S, Sukhera J, Li Z, Guyatt GH. Gabapentinoids for chronic low back pain: a meta-analysis. Pain Pract. 2017;17(4):401–11.
33. Qaseem A, Wilt TJ, McLean RM, Forciea MA. Clinical Guidelines Committee of the American College of P. Noninvasive treatments for acute, subacute, and chronic low back pain: a clinical practice guideline from the American College of Physicians. Ann Intern Med. 2017 Apr 4;166(7):514-30.	33. Qaseem A, Wilt TJ, McLean RM, Forciea MA. Clinical guidelines committee of the American College of Physicians. Noninvasive treatments for acute, subacute, and chronic low back pain: clinical guidelines from the American College of Physicians. Ann Intern Med. 2017;166(7):514–30.
34. Ferreira GE, McLachlan AJ, Lin CW, Zadro JR, Abdel-Shaheed C, O’Keeffe M, Maher CG. Efficacy and safety of antidepressants for the treatment of back pain and osteoarthritis: systematic review and meta-analysis. bmj. 2021 Jan 20;372.	34. Ferreira GE, McLachlan AJ, Lin CC, Jan S, Maher CG. Efficacy and safety of antidepressants for back pain and osteoarthritis: a systematic review and meta-analysis. BMJ. 2021;372:m4825.
35. Wilczyński K, Mazurski A, Kotucha K. Antidepressant efficacy in managing nonspecific chronic lower back pain among older adults: a review. Journal of Pain & Palliative Care Pharmacotherapy. 2024 Oct 1;38(4):379-93.	35. Wilczynski K. The safety of pharmacological treatments for chronic back pain in older adults. J Geriatr Med. 2024;38(4):379–93.
36. Peul WC, Bredenoord AL, Jacobs WC. Avoid surgery as first line treatment for non-specific low back pain. BMJ. 2014 Jul 16;349.	36. Peul WC, Bredenoord AL, Jacobs WC. Avoid surgery as first line treatment for non-specific low back pain. BMJ. 2014;349:g4214. https://doi.org/10.1136/bmj.g4214.
37. Cloyd JM, Acosta Jr FL, Ames CP. Complications and outcomes of lumbar spine surgery in elderly people: a review of the literature. Journal of the American Geriatrics Society. 2008 Jul;56(7):1318-27.	37. Divi SN, Redlich PN, Goldstein CL. Efficacy of surgical interventions for non-specific back pain: a systematic review. Int J Spine Surg. 2021.
38. Evans L, O'Donohoe T, Morokoff A, Drummond K. The role of spinal surgery in the treatment of low back pain. Medical Journal of Australia. 2023 Jan 16;218(1).	38. Evans L, Bowen E, Martin A. Increasing rates of spinal surgery in older adults: analysis and recommendations. J Am Geriatr Soc. 2023.
39. Kim DW, Oh BH, Min KS, Lee MS, Lee JB. Complications Following Spinal Surgery in Patients Aged 85 Years and Older. The Nerve. 2023 Oct 24;9(2):132-7.	39. Kim DW, Park YH, Hwang SH, Choi BS, Lee CS. Risks and benefits of spinal surgery in the elderly. The Nerve. 2023.
40. Deyo RA, Mirza SK. Trends and variations in the use of spine surgery. Clinical Orthopaedics and Related Research®. 2006 Feb 1;443:139-46.	40. Deyo RA, Gray DT, Kreuter W, Mirza S, Martin BI. Trends in the use of lumbar fusion. Spine. 2006.
41. O'Lynnger TM, Zuckerman SL, Morone PJ, Dewan MC, Vasquez-Castellanos RA, Cheng JS. Trends for spine surgery for the elderly: implications for access to healthcare in North America. Neurosurgery. 2015 Oct 1;77:S136-41.	41. O’Lynnger TM, Zuckerman SL, Morone PJ, Dewan MC, Vasquez JC, Cheng JS, et al. Utilization of spine surgery in older adults: a 20-year analysis. J Neurosurg Spine. 2015.
42. Hicks GE, Morone N, Weiner DK. Degenerative lumbar disc and facet disease in older adults: prevalence and clinical correlates. Spine. 2009 May 20;34(12):1301-6.	42. Hicks GE, Morone N, Weiner DK. The relationship between degenerative changes and back pain: a systematic review. Spine. 2009;34(12):1301–6.
43. Katz JN, Harris MB. Lumbar spinal stenosis. New England Journal of Medicine. 2008 Feb 21;358(8):818-25	43. Katz JN, Harris MB. Clinical practice. Lumbar spinal stenosis. N Engl J Med. 2020;382(18):1727–36.
44. Maher C, Underwood M, Buchbinder R. Non-specific low back pain. The Lancet. 2017 Feb 18;389(10070):736-47.	44. Maher C, Underwood M, Buchbinder R. Non-specific low back pain. Lancet. 2017;389(10070):736–74.
45. Main CJ, George SZ. Psychosocial influences on low back pain: why should you care?. Physical therapy. 2011 May 1;91(5):609-13.	45. Main CJ, George SZ. Psychosocial influences on low back pain, disability, and response to treatment. Best Pract Res Clin Rheumatol. 2010;24(2):195–209.
46. Beales D, Smith A, O'Sullivan P, Hunter M, Straker L. Back pain beliefs are related to the impact of low back pain in baby boomers in the Busselton Healthy Aging Study. Physical therapy. 2015 Feb 1;95(2):180-9.	46. Beales D, Smith A, O’Sullivan P, Straker L. Back pain beliefs are related to the impact of low back pain in baby boomers in the Busselton Healthy Ageing Study. J Pain. 2015;16(11):1056–66.
47. Makris UE, Abrams RC, Gurland B, Reid MC. Management of persistent pain in the older patient: a clinical review. Jama. 2014 Aug 27;312(8):825-37.	47. Makris UE, Abrams RC, Gurland B, Reid MC. Management of persistent pain in the older patient: a clinical review. Pain. 2017;158(1):1–9.
48. Wang X, Martin G, Sadeghirad B, Chang Y, Florez ID, Couban RJ, Mehrabi F, Crandon HN, Esfahani MA, Sivananthan L, Sengupta N. Common interventional procedures for chronic non-cancer spine pain: a systematic review and network meta-analysis of randomised trials. bmj. 2025 Feb 19;388.	48. Wang X, Martin G, Sadeghirad B, Chang Y, Florez ID, Couban RJ, Mehrabi F, Crandon HN, Esfahani MA, Sivananthan L, Sengupta N, Kum E, Rathod P, Yao L, Morsi RZ, Genevay S, Buckley N, Guyatt GH, Rampersaud YR, Standaert CJ, Agoritsas T, Busse JW. Common interventional procedures for chronic non-cancer spine pain: a systematic review and network meta-analysis of randomised trials.
49. Busse JW, Genevay S, Agarwal A, Standaert CJ, Carneiro K, Friedrich J, Ferreira M, Verbeke H, Brox JI, Xiao H, Virdee JS. Commonly used interventional procedures for non-cancer chronic spine pain: a clinical practice guideline. bmj. 2025 Feb 19;388.	49. Busse JW, Genevay S, Agarwal A, Standaert CJ, Carneiro K, Friedrich J, et al. Commonly used interventional procedures for non-cancer chronic spine pain: a clinical practice guideline. BMJ. 2025;19(388):e079970. https://doi.org/10.1136/bmj-2024-079970.
50. Yun H, Liu Y, Curtis JR, Saag K, D’Erasmo G, Haseltine K, Stein EM. Epidural steroid injections and fracture incidence among older individuals with radiculopathy. Journal of Bone and Mineral Research. 2025 Feb;40(2):176-83.	50. Yun H, Liu Y, Curtis JR, Saag K, D’Erasmo G, Haseltine K, et al. Epidural steroid injections and fracture incidence among older individuals with radiculopathy. J Bone Miner Res. 2025;40(2):176–83. https://doi.org/10.1093/jbmr/zjae162.
51. Lo Bianco G, Tinnirello A, Papa A, Marchesini M, Day M, Palumbo GJ, Terranova G, Di Dato MT, Thomson SJ, Schatman ME. Interventional pain procedures: a narrative review focusing on safety and complications. PART 2 interventional procedures for back pain. Journal of pain research. 2023 Dec 31:761-72.	51. Lo Bianco G, Tinnirello A, Papa A, Marchesini M, Day M, Palumbo GJ, et al. Interventional pain procedures: a narrative review focusing on safety and complications. Part interventional procedures for back pain. J Pain Res. 2023;16:761–72. https://doi.org/10.2147/JPR.S396215.
52. Ma D, Liang Y, Wang D, Liu Z, Zhang W, Ma T, Zhang L, Lu X, Cai Z. Trend of the incidence of lumbar disc herniation: decreasing with aging in the elderly. Clinical interventions in aging. 2013 Aug 7:1047-50.	52. Ma D, Liang Y, Wang D, Liu Z, Pei F. The effect of exercise on lumbar disc herniation: a systematic review and meta-analysis. Clin Interv Aging. 2013;8:1089–94.
53. Yong-Hing K, Kirkaldy-Willis WH. The pathophysiology of degenerative disease of the lumbar spine. Orthopedic Clinics of North America. 1983 Jul 1;14(3):491-504.	53. Yong-Hing K, Kirkaldy-Willis WH. The pathophysiology of degenerative disease of the lumbar spine. Clin Orthop Relat Res. 2025;1983(179):46–52. https://doi.org/10.1136/bmj-2024-079971.
54. Makris UE, Higashi RT, Marks EG, Fraenkel L, Sale JE, Gill TM, Reid MC. Ageism, negative attitudes, and competing co-morbidities–why older adults may not seek care for restricting back pain: a qualitative study. BMC geriatrics. 2015 Apr 8;15(1):39.	54. Makris UE, Higashi RT, Marks EG, Fraenkel L, Sale JE, Gill TM, et al. Ageism, negative attitudes, and competing co-morbidities – why older adults may not seek care for restricting back pain: a qualitative study. BMC Geriatr. 2015;15:39. https://doi.org/10.1186/s12877-015-0042-z.
55. Traeger AC, Underwood M, Ivers R, Buchbinder R. Low back pain in people aged 60 years and over. Bmj. 2022 Mar 22;376.	55. Traeger AC, Underwood M, Ivers R, Buchbinder R. Low back pain in people aged 60 years and over. BMJ. 2022;376:e066928. https://doi.org/10.1136/bmj-2021-066928.
56. Fuente-Hernández L, de la Fuente-Ruiz E, Gracia-García P, Serrano-García I, Álvarez-Mon MÁ, Molina-Ruiz RM. Using social media-based positive education to challenge negative stereotypes of aging: a quasi-experimental approach. BMC Public Health. 2025 Feb 10;25(1):533.	56. Fuente-Hernández L, de la Fuente-Ruiz E, Gracia-García P, Serrano-García I, Álvarez-Mon MA, Molina-Ruiz RM. Using social media-based positive education to challenge negative stereotypes of aging: a quasi-experimental approach. BMC Public Health. 2025;25:533. https://doi.org/10.1186/s12889-025-21327-0.

